# Unsupervised machine learning model for phenogroup-based stratification in acute type A aortic dissection to identify postoperative acute gastrointestinal injury

**DOI:** 10.3389/fcvm.2024.1514751

**Published:** 2025-01-13

**Authors:** Yuhu Ma, Xiaofang Yang, Chenxiang Weng, Xiaoqing Wang, Baoping Zhang, Ying Liu, Rui Wang, Zhenxing Bao, Peining Yang, Hong Zhang, Yatao Liu

**Affiliations:** ^1^ Department of Anesthesiology and Operation, The First Hospital of Lanzhou University, Lanzhou, Gansu, China; ^2^ Department of Cardiac Surgery, The First Hospital of Lanzhou University, Lanzhou, Gansu, China; ^3^ The First School of Clinical Medicine, Lanzhou University, Lanzhou, China

**Keywords:** acute type A aortic dissection, unsupervised machine learning, phenogroups, random forest, prediction

## Abstract

**Objective:**

We aimed to explore the application value of unsupervised machine learning in identifying acute gastrointestinal injury (AGI) after extracorporeal circulation for acute type A aortic dissection (ATAAD).

**Methods:**

Patients who underwent extracorporeal circulation for ATAAD at the First Hospital of Lanzhou University from January 2016 to January 2021 were included. Unsupervised machine learning algorithm was used to stratify patients into different phenogroups according to the similarity of their clinical features and laboratory test results. The differences in the incidence of perioperative AGI and other adverse events among different phenogroups were compared. Logistic regression was used to analyze the high-risk factors for AGI in each phenogroups and random forest (RF) algorithms were used to construct diagnostic models for AGI in different phenogroups.

**Results:**

A total of 188 patients were included, with 166 males and 22 females. Unsupervised Machine Learning stratified patients into three phenogroups (phenogroup A, B, and C). Compared with other phenogroups, phenogroup B patients were older (*P* < 0.01), had higher preoperative lactate and D-dimer levels, and had the highest incidence of AGI (52.5%, *P* < 0.001) and in-hospital mortality (18.6%, *P* = 0.002). The random forest model showed that the top four risk factors for AGI in phenogroup B were cardiopulmonary bypass time, operation time, aortic clamping time, and ventilator time, which were significantly different from other phenogroups. The areas under the curve (AUCs) for diagnosing postoperative AGI of phenogroup A, B, and C were 0.943 (0.854–0.992), 0.990 (0.966–1.000), and 0.964 (0.899–0.997) using the RF model, respectively.

**Conclusion:**

Phenogroup stratification based on unsupervised learning can accurately identify high-risk populations for postoperative AGI in ATAAD, providing a new approach for implementing individualized preventive and therapeutic measures in clinical practice.

## 1 Introduction

Acute type A aortic dissection (ATAAD) is a life-threatening condition characterized by a tear in the inner layer of the aortic wall, resulting in the separation of the aortic layers and the formation of a true and false lumen (1, 2). The incidence of ATAAD has been increasing annually, and it is associated with high morbidity and mortality rates (3). Surgical intervention is one of the crucial therapeutic modalities for patients with ATAAD (4, 5). However, acute gastrointestinal injury (AGI), a common postoperative complication, severely compromises the surgical outcome and represents a significant contributor to the high postoperative mortality rate in ATAAD patients (6, 7). AGI can manifest as various gastrointestinal complications, such as gastrointestinal bleeding, intestinal obstruction, and ischemic bowel disorders, further exacerbating the postoperative course (8). Low cardiac output, inflammatory responses, surgical trauma, and prolonged extracorporeal circulation are considered the primary risk factors for the development of AGI in ATAAD patients undergoing surgical repair (9). The occurrence of AGI is often insidious, and clinical manifestations lack specificity, frequently leading to delayed diagnosis and treatment, thereby exacerbating the condition (10). Therefore, early identification and intervention for high-risk populations of AGI are crucial for improving the surgical prognosis of ATAAD patients.

Personalized medicine aims to optimize treatment strategies for each individual patient to maximize therapeutic efficacy. Accurate patient stratification is a prerequisite for achieving personalized medicine. The postoperative AGI population in ATAAD patients exhibits high heterogeneity, and some standard treatment protocols have limited efficacy (11). Unsupervised machine learning techniques can perform patient clustering analysis based on multidimensional features (such as demographics, medical history, and laboratory indices), thereby identifying intrinsically similar patient Phenogroups. These approaches have been utilized for Phenogroup clustering in heart failure and sepsis patients to identify the effectiveness of different interventions (12, 13). By analyzing the associations between specific Phenogroups and outcomes or treatment responses, unsupervised machine learning can provide a basis for developing personalized treatment strategies.

Therefore, this study proposes to apply unsupervised machine learning methods to exploit the heterogeneous data of ATAAD patients and identify intrinsic similarities, thereby enabling the recognition of high-risk populations for postoperative AGI. This approach aims to provide guidance for early interventions in this patient population.

## 2 Methods

**Figure 1:**
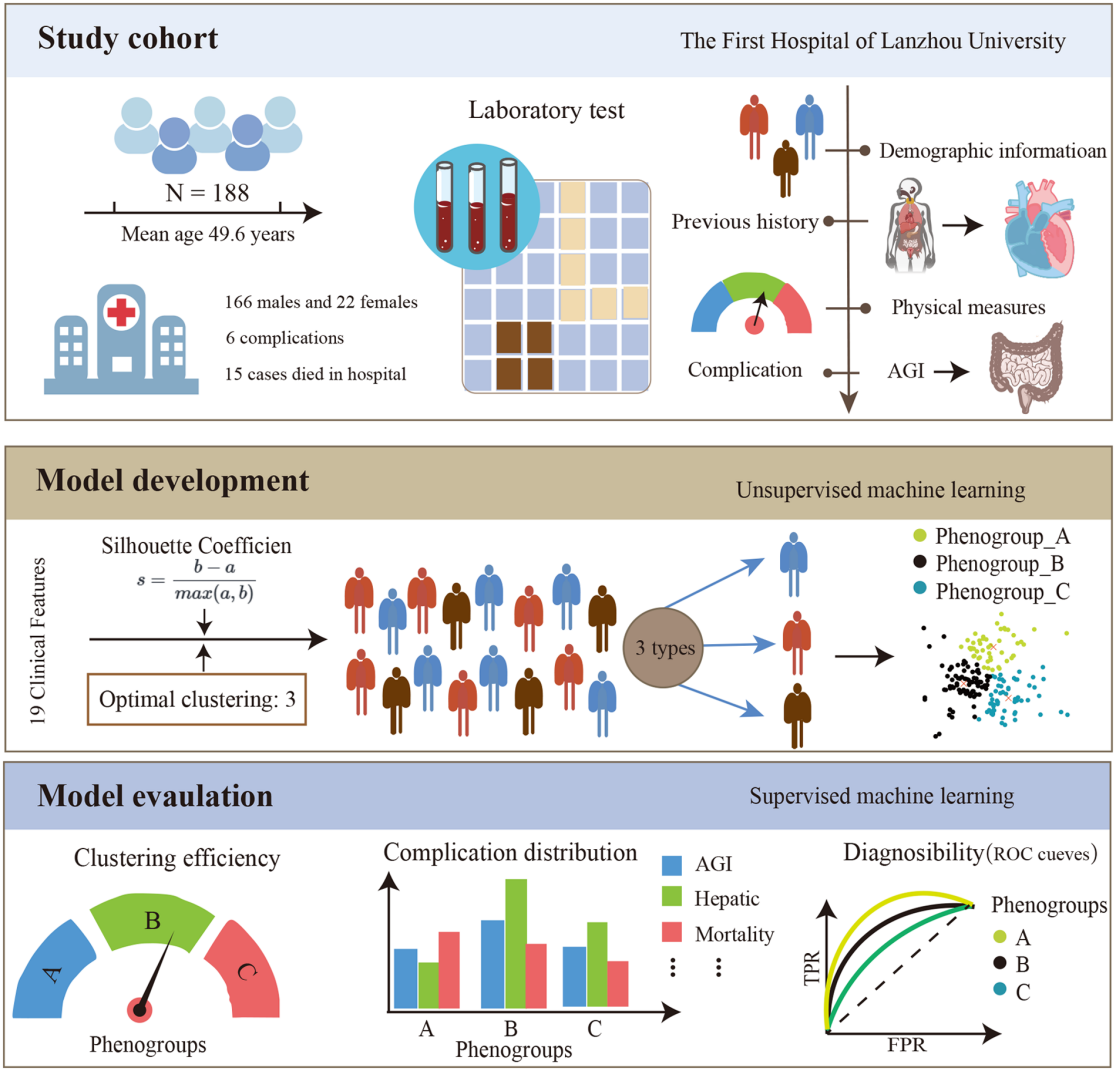
The framework of this study.

Patient studies were conducted according to the guiding principles of the Helsinki Declaration. This study was approved by the Ethics Committee of the First Hospital of Lanzhou University (LDYYLL-2021-422). Due to the retrospective nature of the study, written informed consent was abandoned. The framework of this study is shown in Figure 1.

### 2.1 Patients

Patients hospitalized for ATAAD from January 2016 to January 2021 were identified from the institutional database. Exclusion criteria: (1) Patients with severe hematological, respiratory, or other severe comorbidities; (2) Patients with preoperative acute or chronic gastrointestinal disorders (gastrointestinal hemorrhage, diarrhea, pancreatitis, cholecystitis, cholelithiasis, peptic ulcers, history of abdominal trauma surgery, and gastrointestinal malignancies); (3) Perioperative mortalities and mortalities within 3 days postoperatively; (4) Patients with missing data exceeding 15%.

### 2.2 AGI diagnosis

AGI diagnostic criteria and severity assessment were based on the 2012 AGI guidelines from the European Society of Intensive Care Medicine (14). The severity is graded as follows: Grade 0: Absence of gastrointestinal symptoms. Grade 1: Self-limiting condition, but with a high risk of progression to gastrointestinal dysfunction or failure. Grade 2: Interventional management is necessitated to restore gastrointestinal function (gastrointestinal dysfunction). Grade 3: Persistent gastrointestinal failure despite interventional management (gastrointestinal failure). Grade 4: Acute, life-threatening gastrointestinal insult. Supplementary Table S1 list the evaluation criteria that AGI patients. The AGI grading system was utilized for daily assessment in accordance with standard care during the initial seven days of follow-up. The patients were divided into two groups by the maximum AGI grade: Grade 0 and Grade 1 as the non-AGI group, and Grade ≥2 as the AGI group.

### 2.3 Data collection

Laboratory tests parameters were obtained by reviewing patients' medical records. Which included:

(1) Baseline patient characteristics: gender, age, body mass index (BMI), medical history, and aortic dissection (AD) risk score. (2) Preoperative and postoperative laboratory examinations, including white blood cell and neutrophil percentage (*N*%), as well as serum levels of amylase, total cholesterol, triglycerides, total bilirubin, direct and indirect bilirubin, alkaline phosphatase (ALP), alanine aminotransferase (ALT), aspartate aminotransferase (AST), and γ-glutamyl transferase (γ-GT). (3) Imaging parameters, including the results of computed tomography angiography (CTA) performed upon admission. (4) Records of the surgical procedure, including cardiopulmonary bypass (CPB) time, circulatory arrest time, and intraoperative red blood cell transfusion. Postoperative clinical outcome data: postoperative complications (renal dysfunction, liver dysfunction, and nosocomial infections), intensive care unit (ICU) length of stay, AGI, and in-hospital mortality.

### 2.4 Model construction and evaluation

The K-means clustering algorithm, an unsupervised machine learning technique, was applied to cluster ATAAD patients based on their laboratory test results and clinical baseline information. The K-means algorithm aims to partition n observations into k clusters, minimizing the sum of squared distances between data points and their assigned cluster centroids (15). The algorithm initializes by randomly selecting k cluster centers, assigns each data point to the nearest centroid, and iteratively recalculates the new centroids until convergence is achieved. To determine the optimal number of clusters k, the study evaluated the clustering quality with different *k* values ranging from 2 to 8 based on the silhouette coefficient. The silhouette coefficient measures the ratio of the similarity of a data point to its own cluster compared to its dissimilarity to other clusters, ranging from −1 to 1, with higher values indicating better clustering performance. The *k* value corresponding to the maximum silhouette coefficient and its associated clustering result were selected as the optimal clustering solution.

Based on the optimal clustering outcome, the study analyzed and compared the incidence of AGI and other postoperative complications among different ATAAD patient subgroups. Additionally, univariate logistic regression was employed to identify risk factors associated with postoperative AGI occurrence in each subgroup. The Random Forest algorithm was then utilized to rank the importance of these risk factors and elucidate the relative weights of predictor variables influencing the risk of AGI across different phenogroups.

### 2.5 Statistical analysis

Python (Version 3.9.0) was used for statistical analysis. Continuous variables were reported as the median and interquartile range (IQR) or mean and standard deviation (SD) and were compared using the Mann-Whitney test or Student's *t* test. Categorical data, presented as numbers and frequencies (%), were compared using the chi-square test or Fisher's exact test. A two-sided *P* value < 0.05 indicated that the corresponding difference was statistically significant.

## 3 Results

### 3.1 Patient characteristics

**Figure 2:**
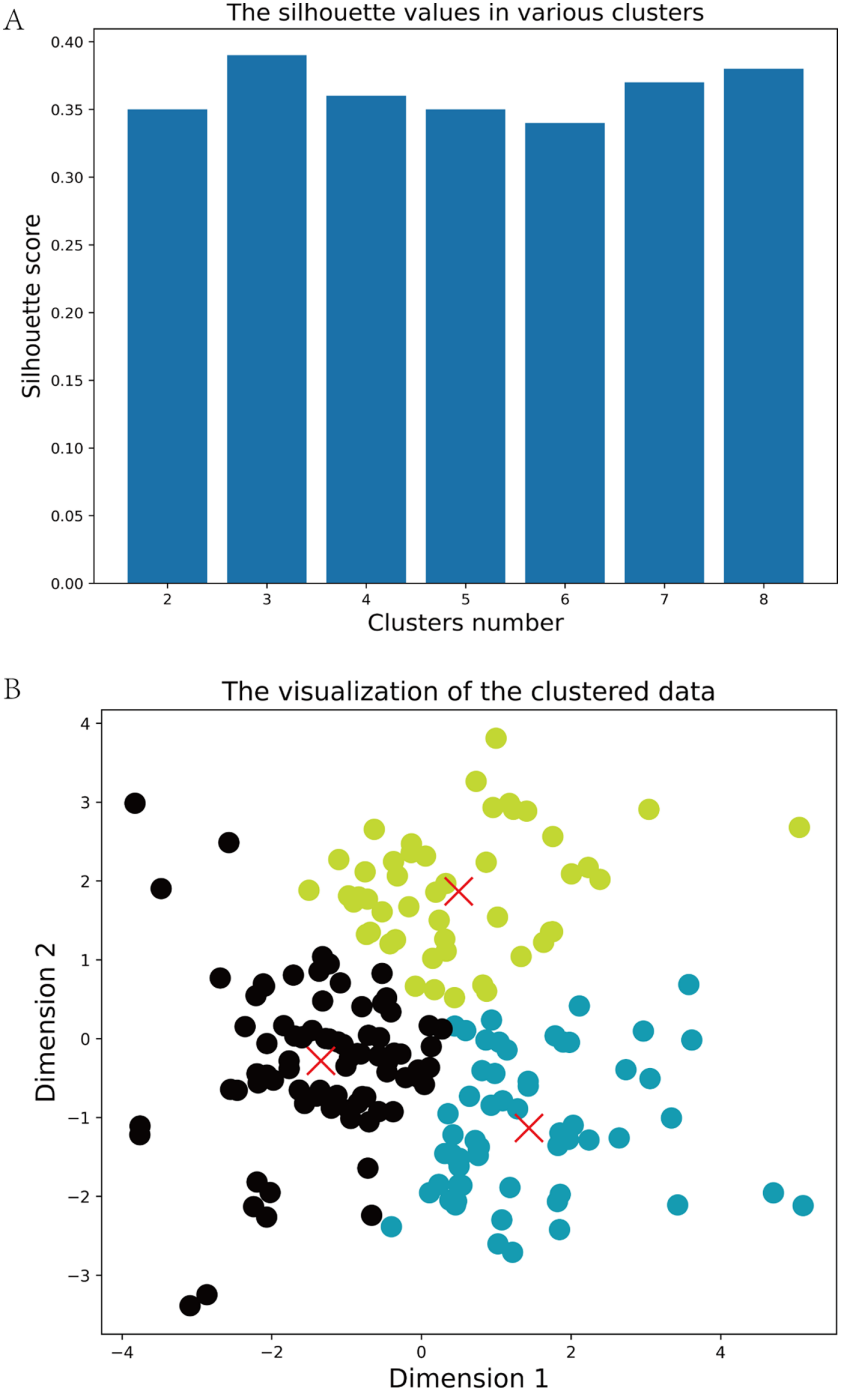
The optimal silhouette coefficient values **(A)** and clustering solution **(B)** in the K-means clustering model. In the panel **(B)**, phenogrophs are visualized using the T-distributed Random Neighborhood Embedding (t-SNE) technique, visualizing the differences between the three phenogroups.

The study included 188 ATAAD patients, with 166 males and 22 females. Postoperatively, 60 patients developed AGI, while 128 patients did not (no-AGI group). Figure 2 illustrates the optimal clustering solution and the silhouette coefficient values in the K-means clustering model. Based on the silhouette coefficient, the optimal number of clusters was determined to be 3, and these phenotypic subgroups were labeled as Phenogroup-A, B, and C. Table 1 presents the baseline characteristics of the three main phenogroups (A, B, and C), comprising 81, 59, and 48 patients, respectively. Compared to the other phenogroups, patients in Phenogroup-B were older and had a higher prevalence of pre-existing heart failure. Additionally, the Phenogroup-B exhibited the highest number of patients with impaired vascular perfusion on computed tomographic angiography (CTA). Laboratory tests revealed significantly elevated lactate and D-dimer levels in Phenogroup-B compared to Phenogroups-A and C.

**Table 1:** Baseline characteristics of the study patients by phenogroups.

Characteristic	Phenogroup A	Phenogroup B	Phenogroup C	*P*
Sex				0.041
Male	15 (18.5%)	4 (6.78%)	3 (6.25%)	
Female	66 (81.5%)	55 (93.2%)	45 (93.8%)	
Age (years)	49.8 (9.31)	54.1 (10.1)	43.9 (7.92)	<0.001
BMI (kg/m^2^)	25.3 (4.03)	24.6 (3.86)	25.8 (4.37)	0.275
Surgical history				0.493
No	79 (97.5%)	58 (98.3%)	45 (93.8%)	
Yes	2 (2.47%)	1 (1.69%)	3 (6.25%)	
AD risk score	1.35 (0.48)	1.53 (0.50)	1.52 (0.50)	0.053
Heart failure				0.004
No	79 (97.5%)	49 (83.1%)	41 (85.4%)	
Yes	2 (2.47%)	10 (16.9%)	7 (14.6%)	
Malperfusion				
Coronary artery				0.021
No	81 (100%)	57 (96.6%)	44 (91.7%)	
Yes	0 (0.00%)	2 (3.39%)	4 (8.33%)	
Renal artery				0.020
No	44 (54.3%)	18 (30.5%)	28 (58.3%)	
Single	31 (38.3%)	34 (57.6%)	15 (31.2%)	
Double	6 (7.41%)	7 (11.9%)	5 (10.4%)	
Superior mesenteric artery				<0.001
No	74 (91.4%)	5 (8.47%)	37 (77.1%)	
Yes	7 (8.64%)	54 (91.5%)	11 (22.9%)	
Aorta abdominalis				<0.001
No	74 (91.4%)	11 (18.6%)	33 (68.8%)	
Yes	7 (8.64%)	48 (81.4%)	15 (31.2%)	
Lactic acid	1.40 (0.61)	2.05 (0.69)	1.75 (0.73)	<0.001
D-dimer assay	8.44 (12.0)	20.9 (27.6)	11.4 (14.8)	0.001
Neutrophils	8.01 (2.59)	9.96 (2.50)	12.0 (3.09)	<0.001
Lymphocytes	1.22 (0.59)	1.11 (0.39)	0.60 (0.25)	<0.001
Monocytes	0.57 (0.28)	0.78 (0.28)	0.39 (0.22)	<0.001
Neutrophils/Lymphocytes	7.64 (3.60)	10.2 (4.56)	23.0 (9.93)	<0.001
Lymphocytes/Monocytes	3.10 (3.14)	1.72 (1.42)	2.34 (2.25)	0.006
PLT	176 (73.5)	142 (41.3)	160 (47.3)	0.004
CRP	35.0 (39.5)	30.7 (30.9)	27.4 (24.1)	0.442

AD, aortic dissection.

### 3.2 Association between phenogroups and postoperative complications

**Figure 3:**
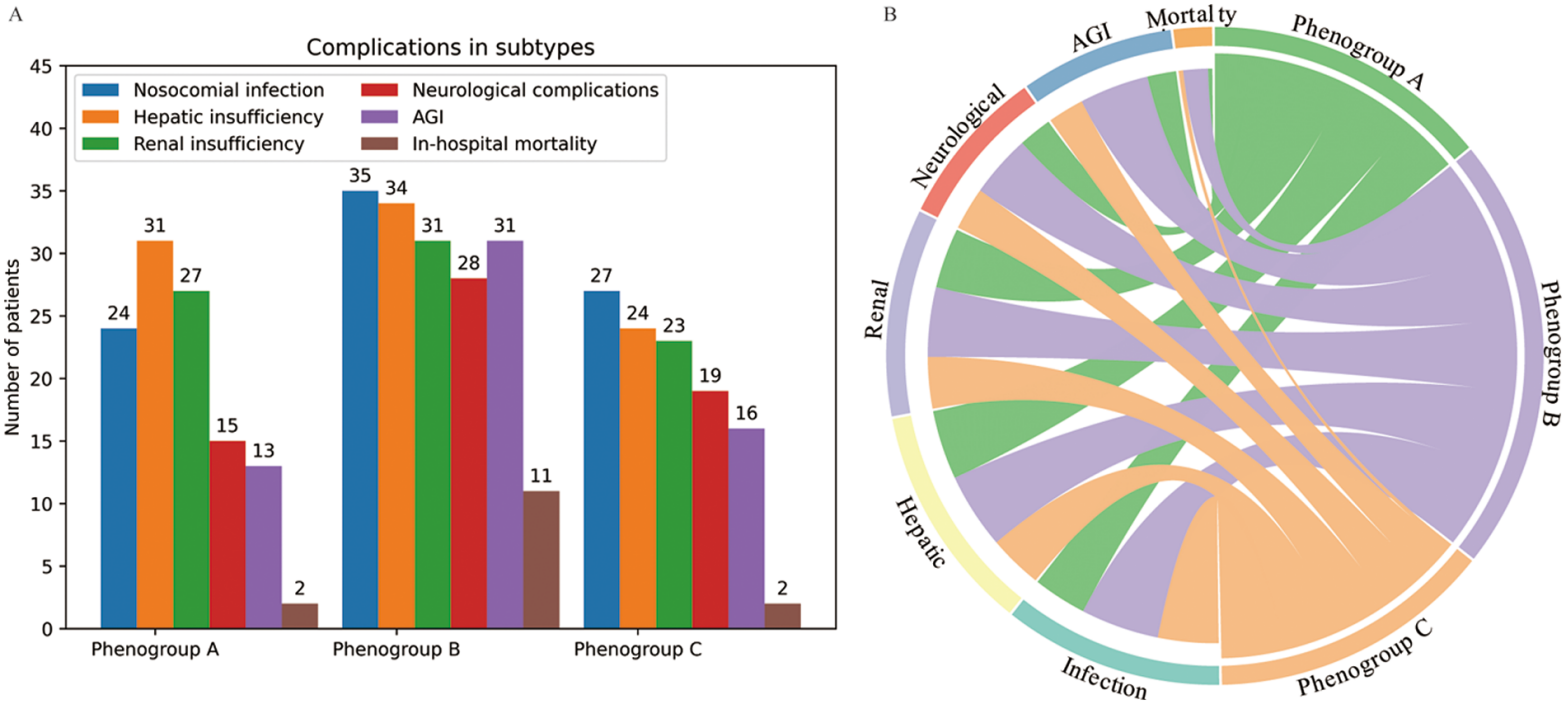
The postoperative complications in the three phenogroups **(A)** and mapping relationship between phenogroups and the complications **(B)**.

The operative time was longest for Phenogroup-B, followed by Phenogroup-A, and shortest for Phenogroup-C (Table 2). However, the cardiopulmonary bypass (CPB) times were not significantly different among the three groups (*P* = 0.169). The aortic cross-clamp time was significantly longer in Phenogroup-B compared to the other two groups (*P* = 0.02). Patients in Phenogroup-B required the highest intraoperative red blood cell transfusion volumes, prolonged mechanical ventilation duration, and longer hospital stays when compared to Phenogroup-A and C. Figure 3 depicts the combined illustration of postoperative complications across the three phenogroups along with the mapping relationship between different phenogroups and the complications. The in-hospital mortality rate was highest in Phenogroup-B (18.6%), followed by Phenogroup-C (4.17%), and lowest in Phenogroup-A (2.47%), the difference was statistically significant (*P* = 0.02). Phenogroup-B had the highest proportion of patients requiring postoperative enteral nutrition (*P* < 0.01) and the highest incidence of infections (59.3%, *P* = 0.001). Additionally, this group was most prone to developing neurological complications. The occurrence of AGI was significantly different among the three groups, with 13, 31, and 16 cases in Phenogroup-A, B, and C, respectively (*P* < 0.001), indicating that Phenogroup-B represents a high-risk phenogroups for AGI.

**Table 2:** Subsequent intraoperative and postoperative outcome characteristics of the three phenogroups.

Outcome	Phenogroup A	Phenogroup B	Phenogroup C	*P*
Operation time (h)	9.73 (2.35)	10.2 (2.82)	9.94 (2.13)	0.586
CPB time(min)	191 (49.4)	218 (97.7)	187 (38.0)	0.020
Aortic clamping time(min)	108 (26.4)	116 (49.0)	104 (24.7)	0.169
Plasma (ml)	1,087 (650)	1,116 (640)	1,039 (668)	0.832
RBC (u)	3.33 (4.55)	5.29 (5.15)	3.35 (3.19)	0.023
Ventilator time (d)	1.84 (1.42)	4.42 (4.83)	2.75 (2.18)	<0.001
ICU time (d)	5.74 (2.73)	9.66 (7.76)	7.81 (3.91)	<0.001
Parenteral nutrition				<0.001
No	74 (91.4%)	38 (64.4%)	40 (83.3%)	
Yes	7 (8.64%)	21 (35.6%)	8 (16.7%)	
Nosocomial infection				0.001
No	57 (70.4%)	24 (40.7%)	21 (43.8%)	
Yes	24 (29.6%)	35 (59.3%)	27 (56.2%)	
Hepatic insufficiency				0.070
No	50 (61.7%)	25 (42.4%)	24 (50.0%)	
Yes	31 (38.3%)	34 (57.6%)	24 (50.0%)	
Renal insufficiency				0.056
No	54 (66.7%)	28 (47.5%)	25 (52.1%)	
Yes	27 (33.3%)	31 (52.5%)	23 (47.9%)	
Neurological complications				0.001
No	66 (81.5%)	31 (52.5%)	29 (60.4%)	
Yes	15 (18.5%)	28 (47.5%)	19 (39.6%)	
AGI				<0.001
No	68 (84.0%)	28 (47.5%)	32 (66.7%)	
Yes	13 (16.0%)	31 (52.5%)	16 (33.3%)	
In-hospital mortality				0.002
No	79 (97.5%)	48 (81.4%)	46 (95.8%)	
Yes	2 (2.47%)	11 (18.6%)	2 (4.17%)	

RBC, red blood cell; CPB, cardiopulmonary bypass.

### 3.3 Associations between phenogroups and inflammatory biomarkers

**Figure 4:**
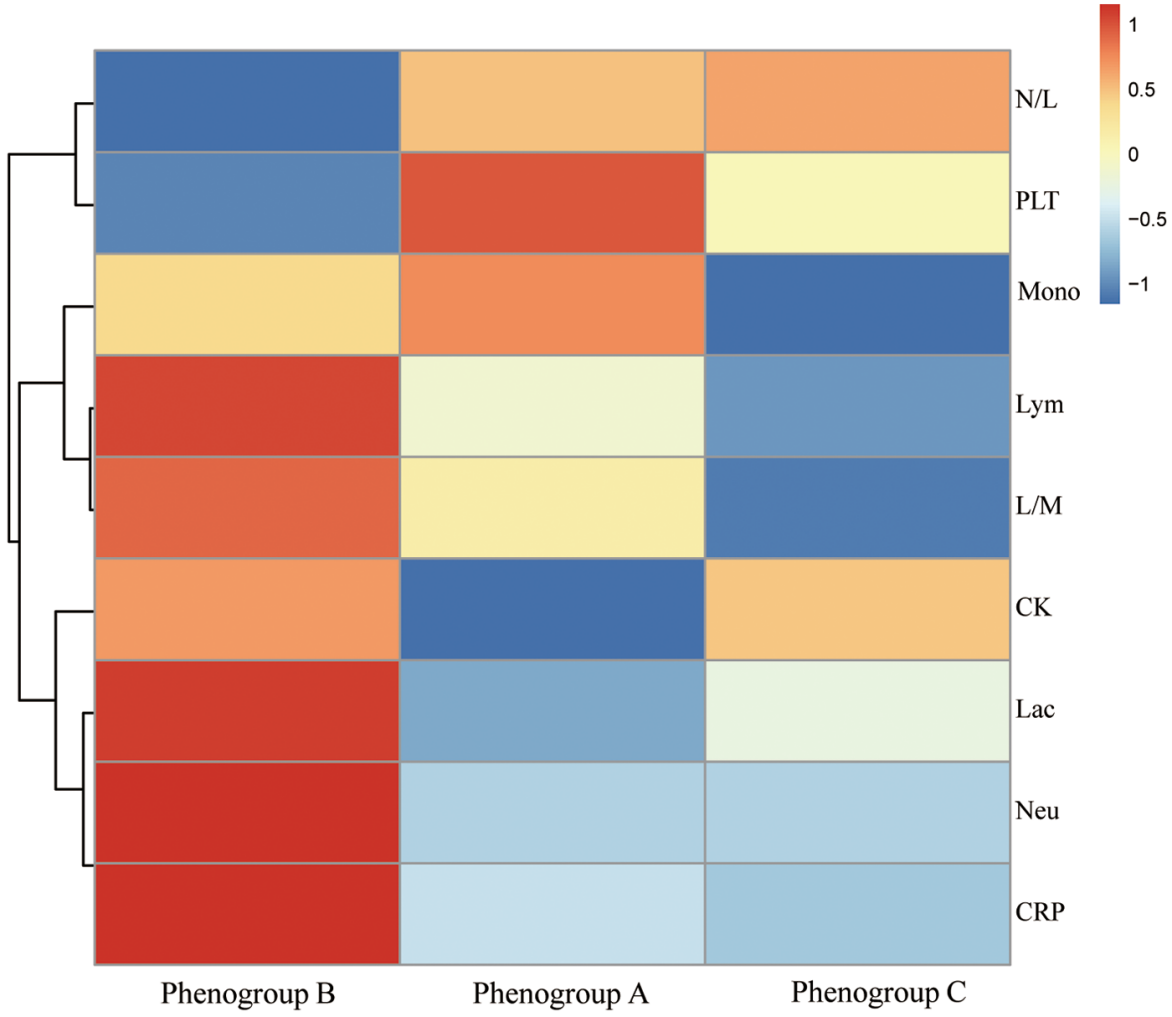
The heat map shows the logarithm of the median of various blood markers with their hierarchical clustering relationship. Neu, neutrophils; Lym, lymphocytes; mono, monocytes; N/L, neutrophils/lymphocytes; L/M, lymphocytes/monocytes; Lc, lactic dehydrogenase; CK, creatine kinase.

Postoperatively, the profiles of inflammatory biomarkers exhibited distinct patterns across the three phenogroups, as shown in Figure 4. Platelet counts declined to varying degrees in Phenogroup-A, B, and C. All three groups demonstrated elevated C-reactive protein (CRP) levels, with the most pronounced upward trend observed in Phenogroup-B, indicating a more robust inflammatory response in this subgroup compared to Phenogroup-A and C. Lactate dehydrogenase (LDH) levels were significantly higher in Phenogroup-B compared to Phenogroup-A and C (*P* = 0.005), while creatine kinase (CK) levels were highest in Phenogroup-C. Overall, no significant differences were observed in neutrophil, lymphocyte, monocyte, neutrophil-to-lymphocyte ratio, or lymphocyte-to-monocyte ratio among the three phenogroups, as presented in Table 3.

**Table 3:** Subsequent intraoperative and postoperative outcome characteristics of the three phenogroups.

Characteristic	Phenogroup A	Phenogroup B	Phenogroup C	*P*
Neutrophils	9.53 (7.56, 11.71)	8.83 (7.28, 11.55)	9.37 (7.705, 11.65)	0.83
Lymphocytes	0.3 (0.18, 0.46)	0.28 (0.19, 0.53)	0.25 (0.2, 0.33)	0.49
Monocytes	0.46 (0.33, 0.64)	0.56 (0.34, 0.73)	0.46 (0.36, 0.59)	0.47
Neu/Lym	30.39 (21.11,49.14)	35.14 (17.72,46.35)	36.09 (24.28, 46.6)	0.51
Lym/Mono	0.54 (.36, 0.98)	0.61 (0.35, 0.98)	0.53 (0.40, 0.75)	0.85
PLT	98 (71, 140)	70 (55, 104)	90 (66.5, 106)	0.002
CRP	98.24 (73, 155.51)	137.05 (101, 204.95)	111.79 (85.79, 136.14)	0.003
Lactic dehydrogenase	475.19 (371.64, 617.24)	613 (422, 1,309)	566.34 (393.88, 895)	0.005
Creatine kinase	1,672 (945, 3,534)	2,674 (1,546, 6,133)	2,921 (1,467.55, 9,087)	0.004

Neu/Lym, Neutrophils/Lymphocytes; Lym/Mono, Lymphocytes/Monocytes.

### 3.4 Risk factor analysis for AGI across different phenogroups

**Figure 5:**
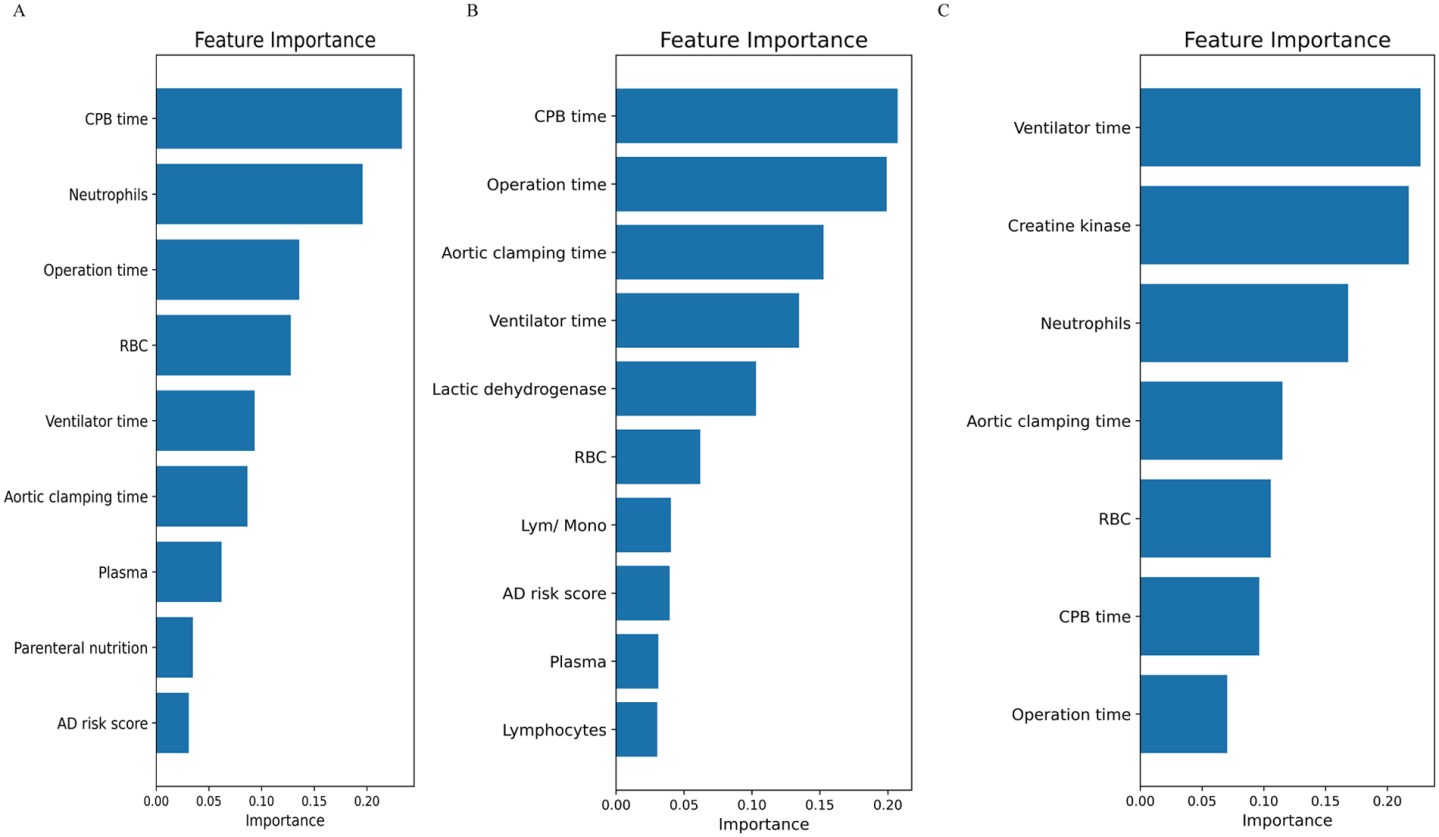
The random forest algorithm was employed to rank the importance of risk factors influencing AGI occurrence. **(A)** phenogroup-A, **(B)** phenogroup-B, **(C)** phenogroup-C.

To evaluate the risk factors contributing to AGI development in the three phenogroups, univariate logistic regression were constructed (Supplementary Table S2). Additionally, the RF algorithm was employed to rank the importance of risk factors influencing AGI occurrence, as shown in Figure 5. For the Phenogroup-B, which exhibited the highest incidence rate of AGI, the top four risk factors identified by the RF analysis were: CPB time, aortic clamping time, operation time, and ventilator time, respectively. This risk factors were distinctly different from the other phenogroups. The ROC curves for diagnosing postoperative AGI of three phenogroups using the RF model are shown in the Supplementary Figure S1. The AUCs for phenogroup-A, B, and C were 0.943 (0.854–0.992), 0.990 (0.966–1.000), and 0.964 (0.899–0.997), respectively. Different risk factors for various phenogroups demonstrate promising diagnostic value for postoperative AGI.

## 4 Discussion

In this study, we applied an unsupervised machine learning algorithm for phenogroups stratification of ATAAD patients undergoing cardiopulmonary bypass, rather than relying solely on individual data points. Three phenogroups were identified, providing motivation for further evaluation of postoperative AGI in ATAAD patients. Patients of different phenogroups exhibited significant differences in the risk and influencing factors of AGI. Notably, phenogroup-B patients had the highest incidence of AGI at 52.5%, significantly higher than the other two phenogroups. This result attests to the accuracy and clinical value of phenogroups stratification in identifying high-risk populations and underscores the potential of machine learning techniques in improving patient stratification and outcomes. This novel approach offers significant advantages for clinical practice, enabling more accurate prediction and management of high-risk patients.

AGI is a severe complication after cardiac surgery, with an extremely high mortality rate (16, 17). The occurrence of AGI is influenced by various perioperative factors, primarily including cardiopulmonary bypass time and the extent of surgical trauma (6). If ischemic hypoperfusion in patients is not diagnosed and addressed promptly in the early stage, it can lead to reduced tissue and organ oxygenation and metabolism, subsequently triggering multiple organ dysfunction and increased mortality (18). Moreover, systemic inflammatory responses constitute a crucial pathophysiological component of postoperative AGI (19). In this study, involving 188 patients with ATAAD undergoing cardiopulmonary bypass, we applied an unsupervised machine learning algorithm to perform phenogroups stratification and evaluated the risk of AGI across different Phenogroups. Utilizing K-means clustering based on clinical and laboratory data, we categorized patients into three distinct phenotypic phenogroups. The results demonstrated significant differences in baseline characteristics among the three phenogroups. The phenogroup-A comprised patients of intermediate age and relatively stable preoperative conditions. Phenogroups-B exhibited the highest risk of AGI, including elderly patients with elevated preoperative lactate and D-dimer levels, 91.5% of patients in this group showed poor mesenteric perfusion. This group also displayed prolonged operative times, increased intraoperative red blood cell transfusion requirements, prolonged mechanical ventilation duration, and extended hospital stays. Computed tomography angiography (CTA) revealed a higher prevalence of preexisting heart failure and impaired vascular perfusion in this phenogroup. Phenogroups-C included younger patients with fewer comorbidities and better preoperative clinical profiles. These differences highlighted the importance of phenotypic heterogeneity in understanding patients' risk profiles. Notably, not only did phenogroups-B patients have the highest incidence of AGI (52.5%), significantly higher than the other two phenogroups, but they also exhibited the highest in-hospital mortality rate. This finding underscored the close association between AGI and poor postoperative outcomes, emphasizing the critical importance of preventing and treating AGI to improve prognosis (20). Such phenogroups stratification is crucial as it allows for personalized, tailored patient care approaches, reflecting the principles of precision medicine. Furthermore, we employed a random RF to analyze the high-risk factors and their weights for AGI in phenogroups-B patients. The top four influencing factors were identified as CPB time, aortic clamping time, operation time, and ventilator time. These factors, to some extent, reflected the magnitude of surgical trauma and the degree of ischemia-reperfusion injury, suggesting a close association between AGI occurrence and surgical trauma as well as ischemia-reperfusion injury (21).

The analysis of postoperative inflammatory biomarkers revealed distinct patterns among the phenogroups. Phenogroup-B showed the most robust inflammatory response, with significantly elevated CRP and LDH levels, indicating a higher degree of systemic inflammation. Previous studies have established that elevated CRP and LDH levels are associated with poor outcomes in gastrointestinal injuries, further corroborating our findings (22, 23). These biomarkers can thus serve as valuable indicators for early intervention and monitoring in high-risk patients. Additionally, the distinct inflammatory profiles suggest that different inflammatory pathways might be involved in AGI pathogenesis among the phenogroups, which could inform the development of targeted therapies.

Traditional risk assessment methods suffer from inherent subjectivity and limitations of single-dimensional analysis. This study pioneered the application of unsupervised machine learning for risk stratification of postoperative AGI in patients with ATAAD. By automatically discovering heterogeneous subgroups based on patients’ multidimensional clinical features, this innovative approach circumvents the need for manual threshold setting and avoids subjectivity. The results revealed significant differences in the incidence of AGI and other postoperative complications across different phenogroups, highlighting the advantages of machine learning in individualized risk assessment. This approach lays the foundation for tailored preventive and therapeutic measures, underscoring its significant clinical translational value. For future research, integrating intraoperative and postoperative dynamic data could further enhance the performance of phenogroup classification and risk prediction models through the development of multi-label classification models for comprehensive evaluation of various postoperative complications. Additionally, extending the application of these models to preoperative assessments could support decision-making for individualized surgical plans and perioperative management strategies, broadening the potential applications of machine learning in perioperative risk assessment (24).

Our study has some limitations. First, the retrospective single-center study design lacks prospective multi-center data, which may limit the extrapolation and generalizability of the results. Second, the sample size is relatively small (188 cases), and after stratification by phenogroups, the sample size of each phenogroups may be further reduced, affecting the statistical power of the analysis. Third, although the high-risk factors for different AGI phenogroups were analyzed, the potential interactions among these factors were not explored, which could aid in the refinement of risk stratification. Fourth, the absence of an external validation cohort may lead to overly optimistic results from the internal validation, and external data is needed to validate the model's generalizability. Finally, although our study provides valuable insights into patient stratification using unsupervised machine learning, the absence of more specific preoperative and intraoperative management strategies (e.g., preoperative restoration of mesenteric perfusion and cardiorespiratory variables) limits the potential for tailoring individualized treatments for high-risk patients identified in this study. Future prospective studies with more comprehensive data collection, including hemodynamic, renal, and perfusion parameters, are necessary to validate our findings and provide more targeted recommendations for clinical practice.

## 5 Conclusions

In conclusion, Phenogroups stratification based on unsupervised learning can accurately identify high-risk populations for postoperative AGI in ATAAD, providing a new approach and basis for implementing individualized preventive and therapeutic measures in clinical practice. This approach has certain innovative value and potential for clinical translation.

## Data Availability

The raw data supporting the conclusions of this article will be made available by the authors, without undue reservation.
