# Supplementary material for: Unsupervised machine learning model for phenogroup-based stratification in acute type A aortic dissection to identify postoperative acute gastrointestinal injury
Source: Front Cardiovasc Med. 2025 Jan 13;11:1514751. doi: 10.3389/fcvm.2024.1514751 (PMC11770000; doi:10.3389/fcvm.2024.1514751)

**Supplementary material**

Table S1. AGI grading system.

| **Grade** | **Definition** | **Rationale** | **Examples** |
| --- | --- | --- | --- |
| **I** | (risk of developing GI dysfunction or failure)-the function of the GI tract is partially impaired, expressed as GI symptoms related to a known cause and perceived as transient. | Condition is clinically seen as occurrence of GI symptoms after an insult, which expectedly has temporary and self-limiting nature. | Postoperative nausea and/ or vomiting during the fifirst days after abdominal surgery, postoperative absence of bowel sounds, diminished bowel motility in the early phase of shock |
| **II** | (gastrointestinal dysfunction)-the GI tract is not able to perform digestion and absorption adequately to satisfy the nutrient and flfluid requirements of the body. There are no changes in general condition of the patient related to GI  problems. | The condition is characterized by acute occurrence of GI symptoms requiring therapeutic interventions for achievement of nutrient and flfluid requirements. This condition occurs without previous GI interventions or is more severe than might be expected in relation to the course of preceding abdominal procedures. | Gastroparesis with high gastric residuals or reflflux, paralysis of the lower GI tract, diarrhoea, intra- abdominal hypertension (IAH) grade I (intra-abdominal pressure (IAP) 12-15 mmHg), visible blood in gastric content or stool. Feeding intolerance is present if at least 20 kcal/kg BW/day via enteral route cannot be reached within 72 h of feeding attempt. |
| **III** | (gastrointestinal failure)-loss of GI function, where restoration of GI function is not achieved despite interventions and the general condition is not improving | Clinically seen as sustained intolerance to enteral feeding without improvement after treatment (e. g. erythromycin, postpyloric tube placement), leading to persistence or worsening of MODS. | Despite treatment, feeding intolerance is persisting-high gastric residuals, persisting GI paralysis, occurrence or worsening of bowel dilatation, progression of IAH to grade II (IAP 15-20 mmHg), low abdominal perfusion pressure (APP) (below 60 mmHg). Feeding intolerance is present and possibly associated with persistence or  worsening of MODS. |
| **IV** | (gastrointestinal failure with severe impact on distant organ function)-AGI has progressed to become directly and immediately life threatening, with worsening of MODS and shock. | Situation when AGI has led to an acute critical deterioration of the general condition of the patient with distant organ dysfunction(s). | Bowel ischaemia with necrosis, GI bleeding leading to haemorrhagic shock, Ogilvie’s syndrome, abdominal compartment syndrome (ACS) requiring decompression. |

Table S2. Univariate and multivariate logistic regression analysis in four Phenogroups.

| **Characteristic** | **Phenogroup A** | | **Phenogroup B** | | **Phenogroup C** | |
| --- | --- | --- | --- | --- | --- | --- |
|  | **OR(95％CI)** | **P** | **OR(95％CI)** | **P** | **OR(95％CI)** | **P** |
| Sex(Male) | 0.434(0.113-1.663) | 0.223 | - |  | - |  |
| Age(Year) | 0.967(0.910-1.028) | 0.289 | 0.957(0.926-1.027) | 0.361 | 1.058(0.974-1.150) | 0.179 |
| BMI | 0.985(0.847-1.146) | 0.852 | 1.115(0.963-1.291) | 0.143 | 1.090(0.944-1.259) | 0.237 |
| Surgical history(Yes) |  |  | - |  | 1.000()0.083-11.931 | 1.000 |
| AD risk score | 3.840（1.118-13.178） | 0.032 | 10.285(3.097-34.157) | ＜0.001 | 0.882(0.265-2.932) | 0.838 |
| Heart failure(Yes) | 5.583(0.326-95.475) | 0.235 | 0.543(0.136-2.169) | 0.388 | 0.288(0.031-2.634) | 0.271 |
| CA malperfusion(Yes) |  |  |  |  | 0.644(0.061-6.740) | 0.714 |
| RA malperfusion(Yes) | 1.022(0.400-2.163) | 0.962 | 1.368(0.579-3.136) | 0.459 | 0.458(0.161-1.299) | 0.143 |
| SMA malperfusion(Yes) | 0.861(0.949-7.813) | 0.849 | 5.000(0.523-47.782) | 0.162 | 3.240(0.806-13.021) | 0.098 |
| Aorta abdominalis(Yes) |  |  | 1.418(0.380-5.285) | 0.603 | 3.571(0.983-12.967) | 0.053 |
| Lactic acid | 1.907(0.714-5.095) | 0.198 | 1.003(0.472-2.129) | 0.993 | 0.544(0.225-1.313) | 0.176 |
| D-dimer assay | 1.023(0.981-1.066) | 0.279 | 0.992(0.972-1.012) | 0.429 | 1.007(0.968-1.047) | 0.722 |
| Neutrophils | 1.100(0.882-1.371) | 0.397 | 1.140(0.921-1.413) | 0.228 | 1.250(1.010-1.548) | 0.040 |
| Lymphocytes | 0.656(0.188-2.282) | 0.508 | 0.489(.125-1.913) | 0.304 | 0.513(0.039-6.701) | 0.611 |
| Monocytes | 5.070(0.613-41.928) | 0.132 | 3.459(0.502-23.775) | 0.207 | 6.548(0.388-110.446) | 0.192 |
| Neutrophils / Lymphocytes | 1.032(0.879-1.212) | 0.879 | 1.114(0.891-1.266) | 0.095 | 1.050(0.986-1.118) | 0.123 |
| Lymphocytes / Monocytes | 0.483(0.215-1.083) | 0.078 | 0.946(0.655-1.366) | 0.770 | 0.599(0.324-1.106) | 0.102 |
| PLT | 0.998(0.989-1.006) | 0.658 | 0.998(0.985-1.010) | 0.755 | 0.992(0.978-1.006) | 0.295 |
| CRP | 1.001(0.987-1.016) | 0.807 | 1.012(0.993-1.030) | 0.129 | 1.011(0.986-1.036) | 0.367 |
| Operation time(h) | 1.784(1.291-2.464) | ＜0.001 | 2.783(1.653-4.685) | ＜0.001 | 1.368(1.000-1.870) | 0.049 |
| CPB time(min) | 1.023(1.009-1.037) | 0.001 | 1.068(1.030-1.106) | ＜0.001 | 1.024(1.003-1.045) | 0.023 |
| Aortic clamping time(min) | 1.028(1.005-1.051) | 0.017 | 1.087(1.036-1.140) | 0.001 | 1.037(1.008-1.066) | 0.012 |
| Plasma（ml） | 1.001(1.000-1.002) | 0.009 | 1.002(1.001-1.003) | 0.003 | 1.001(0.999-1.001) | 0.055 |
| RBC（u） | 1.253(1.057-1.486) | 0.009 | 1.567(1.197-2.051) | 0.001 | 1.428(1.129-1.807) | 0.003 |
| Ventilator time (d) | 1.758(1.208-2.556) | 0.003 | 2.563(1.494-4.394) | 0.001 | 2.896(1.589-5.280) | 0.001 |
| Parenteral nutrition(Yes) | 20.625(3.421-124.337) | ＜0.001 | - |  | - |  |
| Neutrophils | 0.711()0.537-0.941 | 0.017 | 1.045(0.896-1.220) | 0.569 | 0.879(0.667-1.140) | 0.333 |
| Lymphocytes | 0.736(0.281-19.277) | 0.854 | 25.483(1.902-341.264) | 0.014 | 2.527(0.037-171.855) | 0.667 |
| Monocytes | 0.244(0.243-2.439) | 0.230 | 1.040(0.113-9.583) | 0.972 | 12.925(0.743-204.311 | 0.080 |
| Neutrophils / Lymphocytes | 0.993(0.964-1.023) | 0.667 | 0.979(0.951-1.008) | 0.168 | 0.986(0.945-1.028) | 0.519 |
| Lymphocytes / Monocytes | 1.040(0.376-2.872) | 0.939 | 3.155(1.107-8.995) | 0.032 | 0.404(0.067-2.407) | 0.320 |
| PLT | 0.986(0.971-1.001) | 0.074 | 0.986(0.972-1.000) | 0.062 | 0.983(0.964-1.003) | 0.106 |
| CRP | 1.008(0.999-1.017) | 0.064 | 1.006(0.998-1.015) | 0.107 | 1.011(0.997-1.026) | 0.111 |
| Lactic dehydrogenase | 0.999(0.998-1.001) | 0.966 | 1.001(1.000-1.002) | 0.015 | 1.001(0.999-1.002) | 0.219 |
| Creatine kinase | 1.000(0.999-1.000) | 0.393 | 1.000(0.999-1.000) | 0.075 | 1.001(1.000-1.003) | 0.003 |

**Supplementary Figure Legends**

Figure S1. The AUCs for phenogroup A, B, and C.


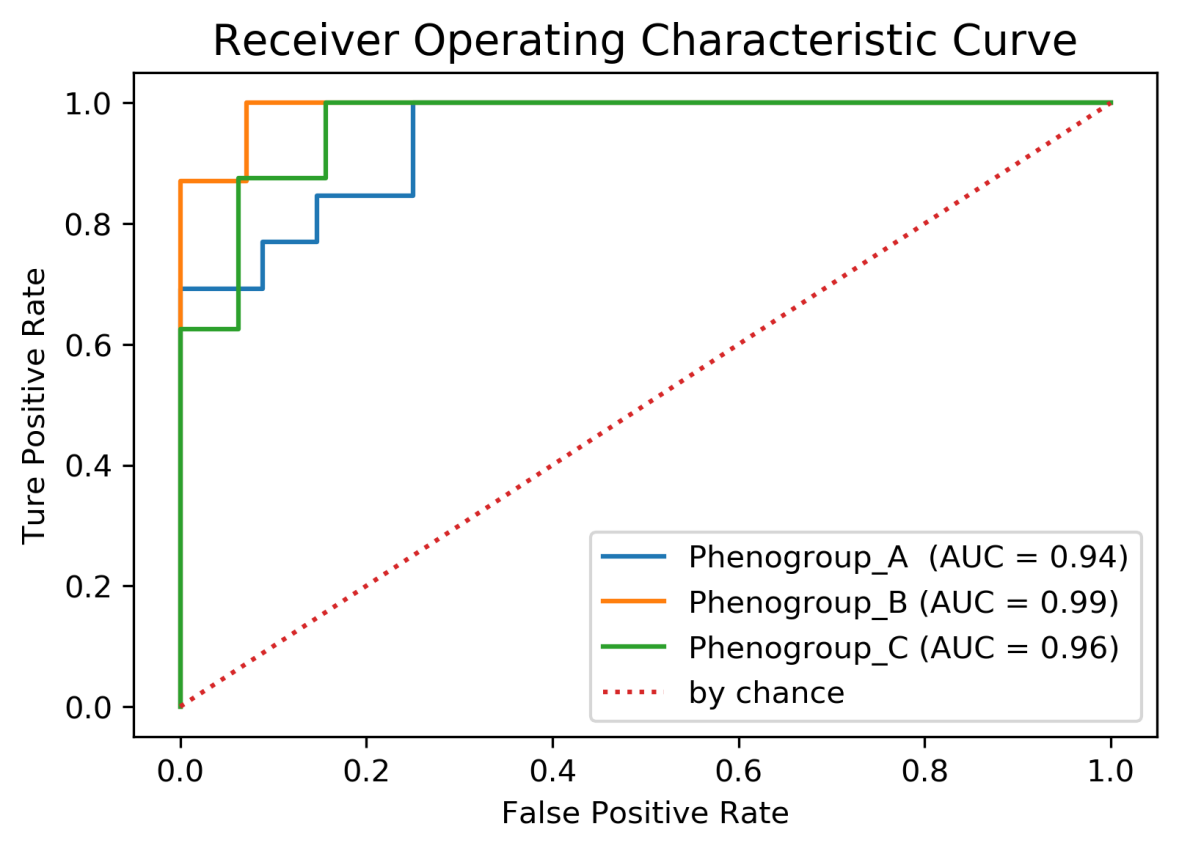

Supplement: Supplementary file 1 [file Datasheet1.docx]
